# Free-energy-based framework for early forecasting of stem cell differentiation

**DOI:** 10.1098/rsif.2019.0571

**Published:** 2019-12-18

**Authors:** H. Suresh, S. S. Shishvan, A. Vigliotti, V. S. Deshpande

**Affiliations:** 1 Department of Engineering, University of Cambridge, Cambridge CB2 1PZ, UK; 2 Department of Structural Engineering, University of Tabriz, Tabriz, Iran; 3 Innovative Materials Laboratory, Italian Aerospace Research Centre, Capua 81043, Italy

**Keywords:** stem cell differentiation, homeostatic mechanics, free-energy model

## Abstract

Commitment of stem cells to different lineages is inherently stochastic but regulated by a range of environmental bio/chemo/mechanical cues. Here, we develop an integrated stochastic modelling framework for predicting the differentiation of hMSCs in response to a range of environmental cues, including sizes of adhesive islands, stiffness of substrates and treatment with ROCK inhibitors in both growth and mixed media. The statistical framework analyses the fluctuations of cell morphologies over approximately a 24 h period after seeding the cells in the specific environment and uses the cytoskeletal free-energy distribution to forecast the lineage the hMSCs will commit to. The cytoskeletal free energy which succinctly parametrizes the biochemical state of the cell is shown to capture hMSC commitment over a range of environments while simple morphological factors such as cell shape, tractions on their own are unable to correlate with lineages hMSCs adopt.

## 1. Introduction

Stem cells have the dual ability to differentiate into various mature cells (such as osteoblasts, chondrocytes, neuroblasts, etc.) that form various tissues, and proliferate to maintain a pool of immature cells that can differentiate when required. The degree and outcome of differentiation are controlled by various extrinsic signals in the stem cell niche that include cell–cell, cell–matrix and cell–soluble cue interactions [1,2]. For example, human mesenchymal stem cells (hMSCs) choose adipogenic over osteogenic lineage when plated at a high cell density, where the extent of cell–cell interactions determines cell fate [3]. The creation of synthetic cellular niches requires careful choices for the extracellular matrix (ECM), and soluble factors (such as growth factors, cytokines and hormones) to best harness the regenerative potential of stem cells [2,4].

While the effect of soluble factors on stem cell lineage commitment and differentiation has been extensively studied, a thorough investigation of the influence of insoluble signals such as ECM rigidity and adhesive properties of the substrate is still ongoing. It is now well known that environmental cues such as microgravity [5] and mechanical cues such as substrate rigidity, substrate curvature, arrangements of micropillars, gratings and wells [6–9] dictate cell fate. Nanoscale physical cues such as nanotubes and nanowires of different pore sizes and spacing, nano-grating, nano-posts and different arrangements of nano-pits [10–14] act at the scale of single focal adhesions to set cell lineage. Advances in nano- and micropatterning have aided in exploring the effect of chemical cues (such as changing the concentration and spacing of adhesive proteins on the substrate [15]) and geometric cues (i.e. confining the cells to adhesive patterns of different shapes and sizes [3,16]) on cell fate. Over the past two decades, numerous experiments have been performed to investigate the effects of mechano/chemo/geometric cues (and their combinations) on the differentiation of multipotent stem cells. At the same time, several models have also been developed and refined using the wealth of information provided by experiments.

One class of models simulate cell shape, cytoskeletal arrangements and focal adhesion formation in cells in response to the physical cues in the ECM, and thereby predict cell differentiation. Qualitative predictions of cell shape-induced differentiation on elastic substrates are obtained using three-dimensional (3D) finite-element models, where relevant subcellular structures are modelled explicitly [17]. Cell–cell interactions are captured through discrete finite-element models (where each cell is a discrete unit that interacts with other cells and the substrate through contact stresses), and the extent of cell deformation is correlated with degree and lineage of differentiation [18]. The spreading of cells on patterned substrates are modelled using particle-based methods, with inter-particle forces chosen to best capture the pinning of cells on substrates by the adhesive islands [19]. While these coarse-grained models provide an intuitive picture of the mechanisms of cell spreading and differentiation, they are often tailored for specific cues.

Another class of models use machine-learning techniques to detect patterns in large volumes of experimental observations that can enable cell fate prediction. The experimental observations can be related to the expression of transcription factors (such as CBFα1 for osteoblasts and PPARγ for adipocytes) [20,21], or various measures of cell shape (such as cell area, aspect ratio, stress-fibre intensity, etc.) [22,23]. However, a frequent output from these models is a complex combination of input variables that seems to correlate strongly with cell fate, but the physical significance of such measures remains poorly understood. Ideas borrowed from statistical mechanics have also been widely used to relate inherently stochastic molecular fluctuations to well-defined macroscopic cell fates [24,25], but such models do not provide insight into cell shape changes associated with the different stages of the differentiation process.

Here, we aim to combine the strengths of the different approaches into one integrated stochastic framework for stem cell differentiation. Recognizing that cells exist in a fluctuating equilibrium with their surroundings, we incorporate fluctuations of cell shape and cytoskeletal structure (rather than at the gene level) so that we can predict both the spreading and differentiation response of hMSCs to chemo-mechanical cues in the ECM. In this study, we present a framework to (i) predict cell differentiation over mechanical and chemical cues and (ii) show the equivalence of different types of chemo-mechanical cues in directing cell lineage commitment and subsequent differentiation.

## 2. Modelling

While it is well established that changes to gene expressions over a period of about one to two weeks dictate the lineage commitment and differentiation fate of hMSCs, more recent studies have indicated that a combination of a large number of cell, nuclear and cytoskeletal morphometrics also provides excellent forecasting of the lineage of hMSCs [22,23]. These morphometrics develop over a period of 1–2 days when the gene expression of cells has not been affected irreversibly by the environment. Here, we apply the recently developed *homeostatic mechanics framework* to predict the distribution of morphological states the cell assumes in the interphase period of its cell cycle, and relate these to the differentiation outcome of hMSCs. The homeostatic mechanics framework has already been shown to successfully capture a range of observations for smooth muscle cells seeded on elastic substrates [26,27] and for myofibroblasts seeded on substrates micropatterned with stripes of fibronectin [28], giving us confidence to investigate its generality in terms of predicting the differentiation of hMSCs in response to a range of environmental cues.

### 2.1. A brief overview of the homeostatic mechanics framework

**Figure 1.**
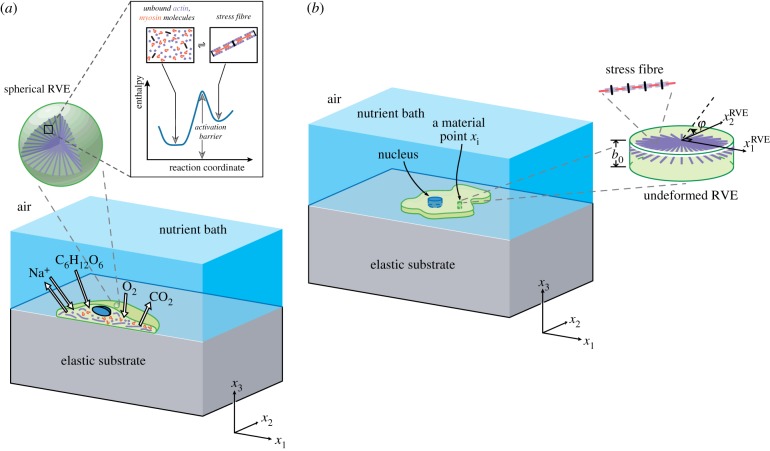
(*a*) An illustration of the cell model employed in the simulations using the homeostatic mechanics framework. The sketch shows a section of a cell on an elastic substrate and exchanging species with the nutrient bath. The inset shows a representative volume element (RVE) of the cell cytoplasm containing polymerized actomyosin stress fibres and the unbound proteins along with the energy landscape that governs the equilibrium of these proteins. (*b*) The 2D approximation of the cell with the 2D RVE. (Online version in colour.)

The homeostatic mechanics framework (see electronic supplementary material, s1.1 for a summary of this framework with further details in [26]) recognizes that the cell is an open system which exchanges nutrients with the surrounding nutrient bath (figure 1*a*). These high-energy nutrient exchanges fuel large fluctuations (much larger than thermal fluctuations) in cell response associated with various intracellular biochemical processes. However, these biochemical processes attempt to maintain the cell in a homeostatic state, i.e. the cell actively maintains itself out of thermodynamic equilibrium [29] by maintaining its various molecular species at a specific average number over these fluctuations that is independent of the environment [30].

More specifically, homeostasis is the ability of a living cell to maintain, via coupled and inter-connected biomechanical processes, the concentration of all internal species at a fixed average value independent of the environment, over all its non-thermal fluctuations (at least over the interphase period of the cell cycle and in the absence of any imposed shock such as starving the cell of nutrients). This implies that over the fluctuations of the cell from any reference state (e.g. typically taken as a cell in suspension as it is easy to compute the free energy for this state) $\langle \Delta N_{i}\rangle =0$, where $\Delta N_{i}$ is the change in the number of molecules of species *i* from its reference value with ⟨x⟩ denoting the average of *x* over the ensemble of states sampled over the non-thermal fluctuations. These fluctuations alter the cell morphology and each morphological microstate has a unique equilibrium Gibbs free energy $G=\sum_{i}\mu_{i}N_{i}$, where $\mu_{i}$ is the chemical potential of species *i*. Using the Gibbs–Duhem relation, we then rewrite this in terms of the reference state as $G=G_{S}+\sum_{i}\mu_{i}^{0}\Delta N_{i}$, where $\mu_{i}^{0}$ is the chemical potential of species *i* in the reference state and $G_{S}$ is the equilibrium Gibbs free energy of an isolated cell in suspension. Upon employing the homeostatic constraint that $\langle \Delta N_{i}\rangle =0$, we have $\langle G\rangle =G_{S}$, i.e. irrespective of the environment, the ensemble average Gibbs free energy is equal to that of the cell in suspension. This is a universal constraint that quantifies the fact that living cells maintain themselves away from thermodynamic equilibrium but yet attain a stationary state.

While the above constraint specifies the average state of the cell, it remains to determine the distribution of states the cell assumes that satisfies this average. Biochemical processes such as actin polymerization and treadmilling provide the mechanisms to explore morphological microstates. Here, we employ the *ansatz* that the observed distribution of cell shapes is that one with the overwhelming number of microstates, i.e. the distribution that maximizes the *morphological entropy* subject to the homeostatic constraint (i.e. $\langle G\rangle =G_{S}$), and any other geometrical constraints such as confinement imposed by patterning adhesive islands on substrates.

Shishvan *et al*. [26] obtained the equilibrium distribution of states that the cell assumes in terms of the Gibbs free energy $G^{\left(j\right)}$ of the morphological microstate (*j*) of the system as

2.1 $$\begin{array}{c} P_{\mathrm{eq}}^{\left(j\right)}=\frac{1}{Z}\exp(-\zeta G^{\left(j\right)}), \end{array}$$

where $Z\equiv \sum_{j}\exp(-\zeta G^{\left(j\right)})$ is the partition function of the morphological microstates, and the distribution parameter *ζ* follows from the homeostatic constraint $\langle G^{\left(j\right)}\rangle \equiv \sum_{j}P_{\mathrm{eq}}^{\left(j\right)}G^{\left(j\right)}=G_{S}$. Thus, 1/*ζ* in (2.1) is referred to as the *homeostatic temperature*, and it uniquely determines the equilibrium distribution of morphological microstates of the cell (also referred to as the *homeostatic ensemble*). We employ Markov chain Monte Carlo to construct a Markov chain that is representative of the homeostatic ensemble. This involves calculation of $G^{\left(j\right)}$ for a given morphological microstate (*j*) and construction of a Markov chain that is representative of the ensemble of states with probability distribution (2.1). Typical Markov chains comprised in excess of 2 million spread states (a detailed overview of the procedure is provided in electronic supplementary material, s1.3).

### 2.2. Gibbs free energy of a morphological microstate

The implementation of the homeostatic mechanics approach described above requires a specific model for the Gibbs free energy of the cell-substrate system in a given morphological microstate (see electronic supplementary material, s1.2 for details). Modelling all the elements of the cell is unrealistic and often not required as specific components are known to strongly respond to different cues. Here, we are interested in investigating the differentiation behaviour of hMSCs due to mechanical cues provided by the substrate stiffness [6] and geometric cues imposed by the size of adhesive islands patterned on substrates [3]. These cues are known to result in significant remodelling of the stress-fibre cytoskeleton and thus here we use a model for the Gibbs free energy developed by Vigliotti *et al.* [31] and subsequently modified in [26–28]. Details of the model including the parameters are given in electronic supplementary material, s1.4 and here we give a brief review.

Single hMSCs are modelled as 2D bodies in the $x_{1}-x_{2}$ plane lying on the surface of an elastic substrate such that the out-of-plane Cauchy stress $\Sigma_{33}=0$ (figure 1*b*). The substrate is modelled as a linear elastic half-space, whereas the cell consists of only three components: a cytoplasm that is modelled as comprising an active stress-fibre cytoskeleton wherein the actin and myosin proteins exist either in unbound or in polymerized states (figure 1*a*), a passive elastic nucleus and elements such as the cell membrane, intermediate filaments and microtubules that are all lumped into a single passive elastic contribution. Thus, the Gibbs free energy of the cell–substrate system in morphological microstate (*j*) follows as $G^{\left(j\right)}\equiv F_{\mathrm{cell}}^{\left(j\right)}+F_{\mathrm{sub}}^{\left(j\right)}=F_{\mathrm{passive}}^{\left(j\right)}+F_{\mathrm{cyto}}^{\left(j\right)}+F_{\mathrm{sub}}^{\left(j\right)}$ (see electronic supplementary material, s1.2 for the details of definitions of the free energies). The cell in its undeformed state (also known as the elastic resting state) is a circle of radius *R*_0_ with a circular nucleus of radius *R*_N_ whose centre coincides with that of the cell with $F_{\mathrm{passive}}=0$ in this undeformed state; see electronic supplementary material, s1.4 for details including the cell parameters used to characterize hMSCs. We emphasize that a cell in suspension is also circular but that state differs from the undeformed state of the cell as the tensile stresses exerted by the stress fibres in the suspended state are balanced by elastic compressive stresses and thus, the cell is deformed in the suspended state.

For a given morphological microstate, the strain distribution within the cell is specified which directly gives the elastic strain energy of the cell ${\hat{F}}_{\mathrm{passive}}$ via a 2D Ogden-type hyperelastic model for both the nucleus and cytoplasm. The stress-fibre cytoskeleton within the cytoplasm is modelled as a distribution of active contractile stress fibres such that at each location *x*_*i*_ within the cell, $\hat{\eta }(\varphi )$ parametrizes the angular concentration of stress fibres over all angles *φ*, while $\hat{n}(\varphi )$ denotes the number of functional units within each stress fibre. Thus, at any *x*_*i*_, there is a total concentration ${\hat{N}}_{b}$ of bound stress-fibre proteins obtained by integrating $\hat{\eta }\hat{n}$ over all orientations *φ*, and these bound proteins are in chemical equilibrium with the unbound stress-fibre proteins (figure 1*a*). The unbound proteins are free to diffuse within the cell, and thus, at equilibrium of a morphological microstate, the concentration ${\hat{N}}_{u}$ of these unbound stress-fibre proteins is spatially uniform. This chemical equilibrium condition along with the conservation of stress-fibre proteins within the cells provides the spatial and angular distributions of stress fibres from which the free energy of the cytoskeleton ${\hat{F}}_{\mathrm{cyto}}$ is evaluated. The tractions that the cell exerts on the substrate induce a Helmholtz free energy ${\hat{F}}_{\mathrm{sub}}$ within the substrate. Then, the total (normalized) free energy of the cell-substrate system in morphological microstate (*j*) follows as ${\hat{G}}^{\left(j\right)}\equiv {\hat{F}}_{\mathrm{passive}}^{\left(j\right)}+{\hat{F}}_{\mathrm{cyto}}^{\left(j\right)}+{\hat{F}}_{\mathrm{sub}}^{\left(j\right)}$ (see electronic supplementary material, s1.5 for details of the normalizations).

### 2.3. Early forecasting of the lineage of human mesenchymal stem cells

A combination of a large number of cell, nuclear and cytoskeletal morphometrics that develop over a period of 1–2 days have been shown to forecast the lineage of hMSCs as measured via gene expressions over a period of about one week [22,23]. While such a morphometric analysis is undoubtedly useful, it has two drawbacks: (i) it requires the measurement and analysis of a large number of morphological metrics and (ii) it provides little insight into the physical phenomena that set the lineage of the cell. A number of studies [3,6,16] have shown that a large fraction of hMSCs remains undifferentiated when the polymerization of the stress-fibre cytoskeleton is inhibited by the addition of re-agents such as cytochalasin-D, Y-27632 or blebbistatin. This combined with the fact that lineage is shown to correlate with cytoskeletal morphometrics suggests that the state of the stress-fibre cytoskeleton can be used to predict cell fate. The stress-fibre cytoskeleton is modelled in detail for each morphological microstate as briefly described above and in detail in electronic supplementary material, s1.2. Of course, a number of cytoskeletal morphometrics can be extracted from the simulations much like in the experiments, but the model has the additional feature that it also quantifies the cytoskeletal free energy ${\hat{F}}_{\mathrm{cyto}}$, which is a succinct scalar parameter that quantifies the biochemical state of the cytoskeleton. Thus, here we attempt to use ${\hat{F}}_{\mathrm{cyto}}$ as the metric to forecast cell fate.

Unlike, deterministic free-energy models [31–33] that treat cells as systems that minimize their free energy, the homeostatic ensemble recognizes that cells exchange nutrients with their environment and thereby maintain a thermodynamic non-equilibrium but nevertheless stationary state that is commonly referred to as the homeostatic state. In this homeostatic state, hMSCs fluctuate over the equilibrium distribution of morphological microstates characterized by (2.1) and thus have a fluctuating ${\hat{F}}_{\mathrm{cyto}}$. Consider the fluctuating response of a hMSC over a time period *T_s_* when it chooses its lineage x, where x, for example, could denote an osteoblast (and then subsequently goes on to differentiate to x over a much longer timescale). Over the time *T*_s_, the average cytoskeletal free energy of the hMSC is given by

2.2 $$\begin{array}{c} {\bar{F}}_{\mathrm{cyto}}=\frac{1}{T_{s}}\int_{0}^{T_{s}}{\hat{F}}_{\mathrm{cyto}}\;dt, \end{array}$$

where time t=0 is an arbitrary reference. We shall assume that the hMSC chooses a lineage x if ${\bar{F}}_{\mathrm{cyto}}$ lies in the range ${\bar{F}}_{x}\pm \Delta {\bar{F}}_{x}$, where ${\bar{F}}_{x}$ and $\Delta {\bar{F}}_{x}$ are values specific to lineage x. In order to estimate the probability of the hMSC choosing a lineage x, we first need to calculate the probability that ${\bar{F}}_{\mathrm{cyto}}$ lies in the range ${\bar{F}}_{x}-\Delta {\bar{F}}_{x}\leq {\bar{F}}_{\mathrm{cyto}}\leq {\bar{F}}_{x}+\Delta {\bar{F}}_{x}$. We hypothesize that cell lineage is typically set over a period of 24–48 h after seeding of hMSCs in a particular environment. Over this period, the cell assumes a large number of morphological microstates. Most of these morphological microstates are correlated with each other, with the cell retaining memory of the history of its state over a timescale of tens of minutes. However, over a longer time period, the cell loses memory and its morphological microstates are decorrelated [34]. Over the period of 24–48 h when the cell chooses its lineage, we assume that hMSCs assume $N_{c}$ decorrelated morphological microstates. This is equivalent to randomly drawing $N_{c}$ morphological configurations from the homeostatic distribution (2.1), and then for large $N_{c}$, the central limit theorem specifies that ${\bar{F}}_{\mathrm{cyto}}$ has a distribution given by the probability density function

2.3 $$\begin{array}{c} \;p({\bar{F}}_{\mathrm{cyto}})=\frac{1}{\sigma }\sqrt{\frac{N_{c}}{2\pi }}\exp\left[\frac{{\left({\bar{F}}_{\mathrm{cyto}}-\mu \right)}^{2}}{2\sigma^{2}/N_{c}^{2}}\right], \end{array}$$

where μ and *σ* are the mean and standard deviation of the homeostatic distribution of ${\hat{F}}_{\mathrm{cyto}}$, i.e. $\mu \equiv \sum_{j}P_{\mathrm{eq}}^{\left(j\right)}{\hat{F}}_{\mathrm{cyto}}^{\left(j\right)}$ and $\sigma^{2}\equiv \sum_{j}P_{\mathrm{eq}}^{\left(j\right)}{\left[{\hat{F}}_{\mathrm{cyto}}^{\left(j\right)}-\mu \right]}^{2}$ with the summations carried out over the entire homeostatic ensemble of morphological microstates (*j*). Given that a hMSC chooses a lineage x if ${\bar{F}}_{\mathrm{cyto}}$ lies in the range ${\bar{F}}_{x}-\Delta {\bar{F}}_{x}\leq {\bar{F}}_{\mathrm{cyto}}\leq {\bar{F}}_{x}+\Delta {\bar{F}}_{x}$, the probability $P_{x}$ of it choosing lineage x is proportional to $P_{x}$ given by

2.4 $$\begin{array}{c} P_{x}=\int_{{\bar{F}}_{x}-\Delta {\bar{F}}_{x}}^{{\bar{F}}_{x}+\Delta {\bar{F}}_{x}}\;p({\bar{F}}_{\mathrm{cyto}})d{\bar{F}}_{\mathrm{cyto}}, \end{array}$$

with the probability $P_{x}$ then defined through a normalizing constant $Z_{L}$ as $P_{x}\equiv P_{x}/Z_{L}$. This normalizing constant ensures that the sum of the probabilities of all lineages (with non-differentiation also treated as a lineage in the context) equals unity. Thus, $Z_{L}\equiv \max\left[1,\sum_{x}P_{x}\right]$ so that if $Z_{L}=1$, the probability of non-differentiation is vanishing and otherwise is given by $1-\sum_{x}P_{x}$. The model for prediction of cell fate thus requires the parameter $N_{c}$ in addition to ${\bar{F}}_{x}$ and $\Delta {\bar{F}}_{x}$ for each lineage x. Typically, these parameters are dependent also on the media in which the hMSCs are cultured and we proceed to present predictions for the differentiation of hMSCs into osteoblasts, myoblasts and adipocytes cultured in growth and mixed media. For a given media, these parameters were calibrated for a given set of cues (e.g. stiffness cues) and then used to make predictions for a different set of cues (e.g. size of adhesive islands). We emphasize that in many cues (e.g. stiffness cues), not all lineages are detected, and therefore, calibration of the full model using experimental data is not always possible. Thus, while calibrating the model here, we lump undetected lineages into the ‘non-differentiation’ category. This approximation does not add any error in the analysis so long as joint probability of differentiation of hMSCs into the detected and undetected lineages is zero. For example, consider the case of hMSCs on a range of substrate stiffness where the hMSCs can differentiate into osteoblasts, myoblasts and adipocytes but the experiment only detects osteoblasts and myoblasts. Since differentiation into adipocytes occurs only for very low stiffness values where the probability of differentiation into osteoblasts and myoblasts is vanishingly small, not accounting for differentiation into adipocytes does not add any errors for predicting the differentiation into osteoblasts and myoblasts.

Although the focus of this study is to predict stem cell differentiation in response to static, 2D chemo-mechanical cues, the methodology outlined here can be readily extended to study differentiation in 3D microenvironments and under cyclic loading conditions. For example, Ristori *et al.* [35] analysed the cytoskeleton reorientation in smooth muscle cells confined to grooved substrates and undergoing cyclic loading. Predictions of the cytoskeletal free energy on substrates with different groove spacing and under different strain rates can be integrated with the differentiation methodology proposed here to estimate the extent and lineage of differentiation.

## 3. Results

We shall consider the response of hMSCs in both growth media and mixed media when seeded on elastic substrates of varying stiffness and on effectively rigid substrates patterned with adhesive islands. The response of the cells in terms of morphometrics over relatively short periods (i.e. 1–2 days) is independent of the differentiation media and thus, the parameters of the hMSCs detailed in electronic supplementary material, s1.4 are not dependent on the media. However, the differentiation outcomes, and thereby, ${\bar{F}}_{x}$ and $\Delta {\bar{F}}_{x}$ are strongly dependent on the media. We thus present results in two steps whereby we first discuss predictions of cell morphometrics and then proceed to discuss predictions of the lineage in the two different media.

### 3.1. Response on elastic substrates

**Figure 2.**
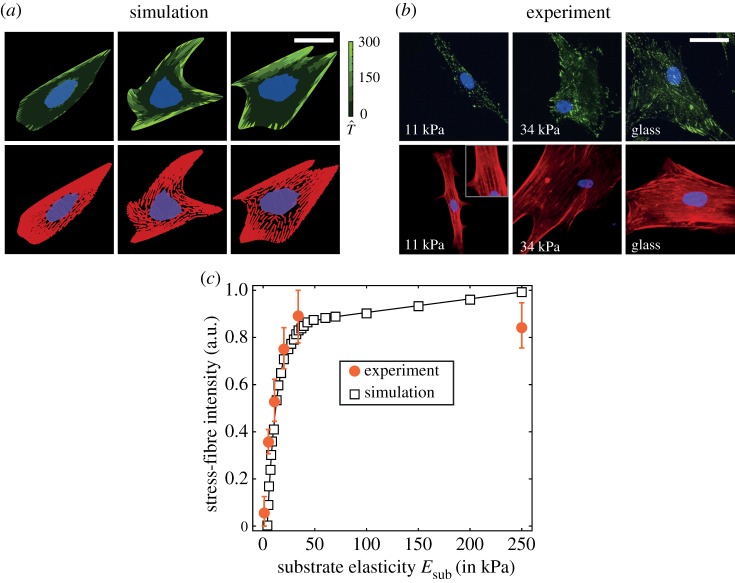
(*a*) Predictions from simulations and (*b*) observations from Engler *et al.* [6] of hMSCs seeded on elastic substrates uniformly coated with collagen. In the experimental immunofluorescence images, the focal adhesions are coloured green, actin red and nucleus blue and purple, and a similar scheme is followed in the predictions, with the focal adhesions parametrized by the magnitude of the normalized traction $\hat{T}$ (see electronic supplementary material, s1.5.1 for details of the method used to construct immunofluorescence-like images from the simulated results). Scale bar, 20 µm. (*c*) Predictions of stress-fibre intensity as a function of substrate stiffness compared with observations from Zemel *et al.* [36] for hMSCs seeded on glass substrates. In the simulations, we use total stress-fibre concentration ${\hat{N}}_{b}\equiv 1-{\hat{N}}_{u}$ to parametrize the stress-fibre intensity. (Online version in colour.)

The response of cells on elastic substrates is recorded through a range of observables, all of which exhibit large variations but with trends clearly emerging when the statistics of these observables are analysed. This motivates our choice of the statistical homeostatic modelling framework, in which, just as in the experimental system, observables fluctuate while trends (and understanding) emerge once these observables are viewed statistically. Figure 2*a* shows representative images of cell morphologies on substrates with three contrasting stiffness $E_{\mathrm{sub}}$ (while glass has a stiffness $E_{\mathrm{sub}}\approx 50\;\mathrm{GPa}$, the effective stiffness experienced by the cell is much lower due to the intermediate ECM, and thus following Engler *et al*. [6], we take $E_{\mathrm{sub}}=70\;\mathrm{kPa}$ for glass as representing the effective stiffness of the ECM). The predictions have been presented alongside the observations (figure 2*b*) of Engler *et al*. [6], replicating the immunofluorescence staining used in experiments whereby the stress fibres are shown in red, the focal adhesions in green and the nucleus in purple and blue (see electronic supplementary material, s1.5.2 for details of the procedure used to translate the model predictions to such images). Overall, the cell morphologies and distributions of cytoskeletal and focal adhesion proteins are similar to the experimental observations. Stress-fibre polymerization, focal adhesion formation, cell area and aspect ratio increase with increasing substrate stiffness, in line with a wide variety of observations [6,8,37]. The stress-fibre intensity captures the extent of stress-fibre polymerization in cells in response to cues in the microenvironment. Figure 2*c* shows remarkable agreement between the myosin fibre intensity measured by Zemel *et al.* [36] (electronic supplementary material, figure S3 *therein*), and the predicted total concentration ${\hat{N}}_{b}\equiv 1-{\hat{N}}_{u}$ of bound stress-fibre proteins as a function of substrate stiffness (see electronic supplementary material, s1.2.1 for details of the definitions).

**Figure 3.**
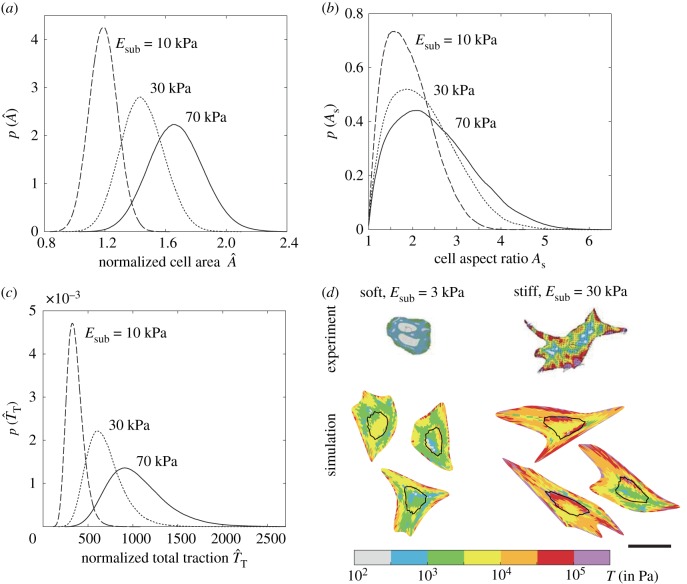
Predictions of the probability density functions of three typically reported observables for hMSCs seeded on elastic substrates uniformly coated with collagen. Distributions of (*a*) normalized cell area $\hat{A}$, (*b*) cell aspect ratio *A*_s_ and (*b*) normalized total traction ${\hat{T}}_{T}$ for three selected substrate stiffness $E_{\mathrm{sub}}$ in each case. (*d*) Comparisons between measurements [37] and predictions of the distributions of tractions *T* exerted by cell on elastic substrates of stiffness $E_{\mathrm{sub}}=3\;\mathrm{kPa}$ and 30 kPa (outline of nucleus shown as a black line). In each case, we show three simulated configurations we randomly selected from the computed homeostatic distribution. The scale bar in (*d*) = 30 µm. (Online version in colour.)

However, as eluded to above, selected observations of cell morphologies are highly variable with understanding emerging from the statistics of observations. Recalling that typically reported observations include distributions of cell area, aspect ratio and total tractions exerted by the cell on substrates, we include in figure 3*a–c* predictions of these observables. These predictions are presented in terms of probability density functions of the normalized area $\hat{\;A}$ (area of the deformed cell normalized by area of the cell in its elastic resting state *A*_0_), aspect ratio $A_{s}$ (ratio of the lengths of the major to the minor axis of the best-fit ellipse to the cell) and the normalized total tractions ${\hat{T}}_{T}$ (see electronic supplementary material, s1.5.1 for details of the definitions). With increasing substrate stiffness, not only do the means of cell area, aspect ratio and total tractions increase, but so does the spread in these observables (i.e. the probability density functions are less peaked with increasing *E*_sub_).

Tractions exerted by hMSCs on elastic substrates have been experimentally established, and we show in figure 3*d*, predictions (for three high probability cell configurations) of the normalized traction distributions $\hat{T}$ (see electronic supplementary material, s1.5.1 for definition) alongside the corresponding measurements from Guvendiren & Burdick [37]. Regions of higher tractions are significantly fewer on the lower stiffness substrates, but also typically occur along the cell periphery in both the observations and predictions. In general, the agreement between model predictions of cell morphometrics and experimental observations gives confidence to attempt to employ it to aid forecasting of cell lineage. However, while substrate stiffness does affect cell response, it is clear that given the significant overlap in the distribution of observables (figure 3*a–c*), it is unlikely that these observables can be directly used to predict cell lineage.

### 3.2. Response of cell on adhesive islands

**Figure 4.**
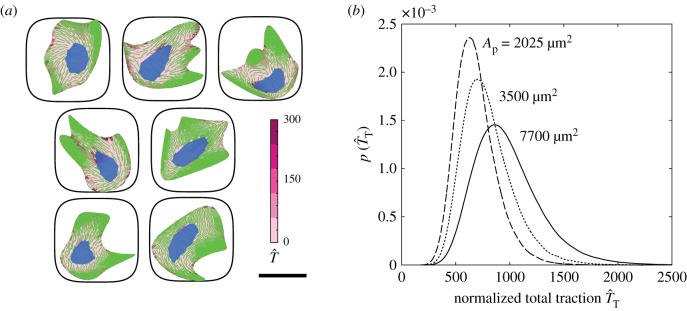
(*a*) A selection of seven cell configurations selected from the homeostatic ensemble for hMSCs seeded on substrate patterned with adhesive islands of area $A_{p}=2025\;\;\mu m^{2}$. The predictions are shown as combined immunofluorescence-like images showing stress fibres (green), focal adhesions (pink) and nucleus (blue). Focal adhesions are parametrized by the magnitude of the normalized traction $\hat{T}$. Scale bar, 30 µm. (*b*) Prediction of the probability density function of normalized total traction ${\hat{T}}_{T}$ for hMSCs seeded on adhesive islands of selected areas *A*_p_. (Online version in colour.)

A selection of highly probable cell configurations of hMSCs on a square adhesive islands of area $A_{p}=2025\;\mu m^{2}$ are included in figure 4*a* (these islands are patterned on PDMS substrates with $E_{\mathrm{sub}}=4\;\mathrm{MPa}$, which is assumed to be effectively rigid, and thus predictions shown correspond to substrates with stiffness $E_{\mathrm{sub}}=70\;\mathrm{kPa}$). These predictions are shown as combined immunofluorescence-like images showing stress fibres (green), focal adhesions (pink) and nucleus (blue). Tractions exerted by hMSCs are considered to be a strong indicator of the lineage they adopt [8,37] and we include predictions of the probability density functions of the normalized total tractions ${\hat{T}}_{T}$ in figure 4*b* for a range of areas *A*_p_ of the square adhesive islands. Again, there is a significant overlap in the traction distributions for the different island sizes, but in general, the tractions that cells exert decrease with decreasing *A*_p_, and this is generally thought to indicate a preference for differentiation into adipocytes over osteoblasts.

### 3.3. Differentiation in growth media

In a landmark experiment, Engler *et al*. [6] established the influence of substrate elasticity on stem cell lineage commitment. Their experiments were performed in a growth medium which encouraged differentiation into osteoblasts, myoblasts and maybe adipocytes depending on substrate stiffness, although the experiments reported in Engler *et al.* [6] did not directly report commitment to adipocytes. Thus, here we only focus on the differentiation of hMSCs to osteoblasts, myoblasts and set ${\bar{F}}_{x}=2.59$ and $\Delta {\bar{F}}_{x}=0.14$ for osteoblasts, while for myoblasts, we set ${\bar{F}}_{x}=1.87$ and $\Delta {\bar{F}}_{x}=0.05$ with $N_{c}=15$.

**Figure 5.**
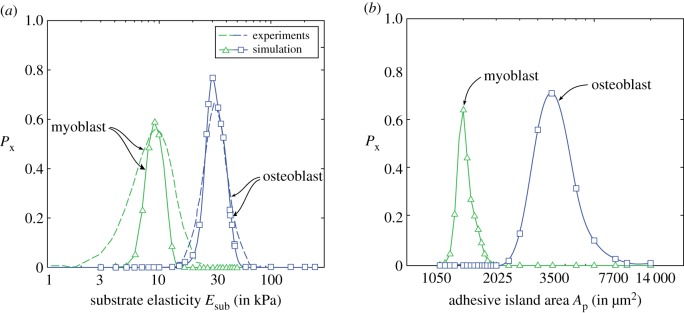
(*a*) Predictions of the variation of differentiation fraction $P_{x}$ for hMSCs seeded on elastic substrates with stiffness $E_{\mathrm{sub}}$ in growth media and compared with measurements from Engler *et al*. [6]. (*b*) Corresponding predictions of $P_{x}$ for hMSCs seeded on substrate patterned with adhesive islands of area *A*_p_ and seeded in growth media. The hMSCs in growth media are assumed to differentiate into osteoblasts, myoblasts or remain undifferentiated. (Online version in colour.)

Predictions of the fraction $P_{x}$ of the hMSCs differentiated into osteoblasts and myoblasts as a function of the substrate stiffness $E_{\mathrm{sub}}$ are included in figure 5*a* alongside measurements from Engler *et al*. [6]. Excellent agreement is obtained, with osteoblasts favoured on the stiffer substrates. We note that even for *E*_sub_ = 30 kPa where the probability of differentiation into osteoblasts peaks (and equally at *E*_sub_ = 10 kPa where the probability of differentiation into myoblasts is a maximum), the differentiation fraction $P_{x}\neq 1$, i.e. the model predicts that at *E*_sub_ = 30 kPa, approximately 20% of the hMSCs remain undifferentiated much like the measurements. The excellent agreement between predictions and observations is of course partially related to the fact that ${\bar{F}}_{x}$ and $\Delta {\bar{F}}_{x}$ for myoblasts and osteoblasts are calibrated against the differentiation data of Engler *et al*. [6]. However, the strength of the approach is that the model can now be used to predict the differentiation response for hMSCs on adhesive islands and these predictions are included in figure 5*b*. While no data exist to date for the differentiation of hMSCs in growth media seeded on adhesive islands, our model suggests a preference for osteoblasts on larger islands and myoblasts on smaller islands. The propensity for differentiation of hMSCs into osteoblasts on larger islands is known at least for hMSCs cultured in mixed media (as will be discussed subsequently), but here our predictions suggest that the mechanical cues from substrate stiffness and geometric cues from sizes of adhesive islands can have similar effects on the differentiation response of hMSCs in growth media.

**Figure 6.**
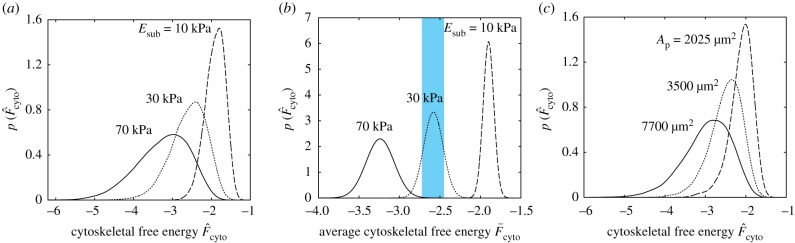
Predictions of the probability density function of (*a*) the cytoskeletal free energy ${\hat{F}}_{\mathrm{cyto}}$ for hMSCs seeded on substrates of selected stiffness $E_{\mathrm{sub}}$ and (*b*) the corresponding distributions of the average cytoskeletal free energy ${\bar{F}}_{\mathrm{cyto}}.$ In (*b*), we have indicated the band (blue) of average cytoskeletal free energies over which hMSCs are assumed to differentiate into osteoblasts in growth media. (*c*) Predictions of the probability density functions of ${\hat{F}}_{\mathrm{cyto}}$ for hMSCs seeded on substrates patterned with adhesive islands of area *A*_p_. (Online version in colour.)

To understand the equivalency of these different cues, we first examine in further detail the differentiation predictions on elastic substrates. We have assumed that differentiation is set by the distribution of the cytoskeletal free energy ${\hat{F}}_{\mathrm{cyto}}$. Predictions of the probability density functions of ${\hat{F}}_{\mathrm{cyto}}$ for hMSCs on substrates with three selected stiffness are included in figure 6*a*. As discussed in [26], higher substrate stiffness allows cells to exert larger tractions without a significant energy penalty, and the probability of cells to adopt configurations with larger cell areas, aspect ratios and higher levels of stress-fibre polymerization increases. A direct consequence of the high level of stress-fibre polymerization is lowering of the cytoskeletal free energy as seen in figure 6*a*. These distributions then via (2.3) give the distribution of the cytoskeletal free energy ${\bar{F}}_{\mathrm{cyto}}$ that cells assume over the 24–48 h period after seeding during which they set their lineage. Predictions of the probability density functions of ${\bar{F}}_{\mathrm{cyto}}$ are included in figure 6*b* for the substrate stiffness employed in figure 6*a*. These distributions are relatively well separated, suggesting that if the differentiation of hMSCs was set by this average cytoskeletal free energy, their response on these three different substrates would vary substantially. In figure 6*b*, we have marked the range ${\bar{F}}_{x}\pm \Delta {\bar{F}}_{x}$ for the differentiation into osteoblasts in growth media. Clearly, a large fraction of hMSCs seeded on the *E*_sub_ = 30 kPa will differentiate into osteoblasts as seen in figure 5*a*, with a very small fraction of cells on *E*_sub_ = 70 kPa also differentiating into osteoblasts as there is a small overlap in the distribution of ${\bar{F}}_{\mathrm{cyto}}$ on *E*_sub_ = 70 kPa with the differentiation range ${\bar{F}}_{x}\pm \Delta {\bar{F}}_{x}$ for osteoblasts. However, there is no overlap with hMSCs on *E*_sub_ = 10 kPa with no differentiation into osteoblasts expected for cells seeded on such soft substrates. Thus, the propensity of hMSCs to differentiate into osteoblasts when seeded on substrates with stiffness $E_{\mathrm{sub}}\approx 30\;\mathrm{kPa}$ is directly related to the fact that their average cytoskeletal free energy is in the correct range: for higher stiffness substrates, ${\bar{F}}_{\mathrm{cyto}}$ is too low due to the higher levels of stress-fibre polymerization, while on softer substrates, ${\bar{F}}_{\mathrm{cyto}}$ is too high due to the significantly reduced levels of stress-fibre polymerization.

Recall that with increasing substrate stiffness, the cytoskeletal free energy decreases, and this is associated with an increase in cell area. Seeding cells on rigid substrates patterned with adhesive islands can restrict the spreading of cells and thereby have a similar effect on the cytoskeletal free energy by constraining stress-fibre polymerization via this geometric cue rather than the stiffness cue. Predictions of the distribution of ${\hat{F}}_{\mathrm{cyto}}$ are included in figure 6*c* for selected adhesive island areas *A*_p_ with cytoskeletal free energy again decreasing with increasing *A*_p_ (for $A_{p}>14\;000\;\;\mu m^{2}$, the adhesive islands do not restrict cell spreading and the results converge to the *E*_sub_ = 70 kPa case discussed above). The consequences are therefore similar to the stiffness cues with hMSCs differentiating into osteoblasts for intermediate island areas.

### 3.4. Differentiation in mixed media

**Figure 7.**
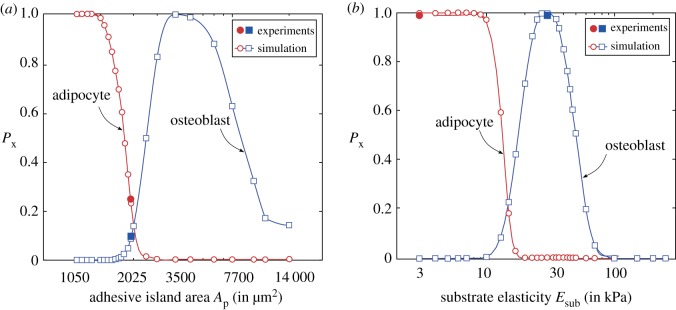
Predictions of the variation of differentiation fraction $P_{x}$ for hMSCs seeded on (*a*) substrates patterned with adhesive islands of area $A_{p}$ and (*b*) elastic substrates with stiffness $E_{\mathrm{sub}}$ in mixed media. The hMSCs in mixed media are assumed to differentiate into osteoblasts, adipocytes or remain undifferentiated. Experimental measurements for an island of area $A_{p}=2025\;\;\mu m^{2}$ from McBeath *et al*. [3] and substrates of stiffness *E*_sub_ = 3 and 30 kPa from Guvendiren & Burdick [37] are included in (*a*) and (*b*), respectively. (Online version in colour.)

In mixed media, hMSCs differentiate into osteoblasts and adipocytes. We keep ${\bar{F}}_{x}=2.59$ unchanged for osteoblasts and increase $\Delta {\bar{F}}_{x}=0.37$ while we choose ${\bar{F}}_{x}=1.61$ and $\Delta {\bar{F}}_{x}=0.44$ for adipocytes. These values are again chosen in order to obtain agreement with measurements for hMSCs seeded on adhesive islands in mixed media [3]. Predictions of the differentiation fraction $P_{x}$ for adipocytes and osteoblasts as a function of the area *A*_p_ of the adhesive islands (atop stiff substrates with stiffness $E_{\mathrm{sub}}=70\;\mathrm{kPa}$) and the corresponding predictions of $P_{x}$ as a function of substrate stiffness $E_{\mathrm{sub}}$ are included in figure 7*a,b*, respectively. The experimentally measured differentiation fraction from McBeath *et al*. [3] for an island size $A_{p}=2025\;\;\mu m^{2}$ and from Guvendiren & Burdick [37] for cells cultured on substrates of stiffness $E_{\mathrm{sub}}=3$ and 30 kPa included in figure 7*a,b* confirm the fidelity of the predictions. Importantly, the equivalency of the stiffness and geometric cues seen for growth media also carries forward to mixed media, where we now see an increased tendency for differentiation into adipocytes at either lower adhesive island areas or lower substrate stiffness.

Overall, the reason for this equivalency is as discussed above: adipocytes are favoured when the average cytoskeletal free energy is higher and this occurs either by restricting cell spreading via the island size or on low stiffness substrates where the large tractions result in an energy penalty from the substrate which prevents cell spreading and enhances the cytoskeletal free energy.

## 4. Discussion

The equivalency of the cues discussed above, i.e. appropriately controlling adhesive island area can have an effect similar to substrate stiffness on hMSC differentiation, seems to suggest that observable morphometrics such as cell area, aspect ratio, etc., might correlate with the lineage the hMSCs adopt. In fact, it has been suggested [38] that changes to cell shape may be transduced into regulatory signals that govern cell fate. However, it is clearly seen from the distributions (figure 3*a–c*) that while cues such as substrate stiffness and island area do affect observables such as cell shape, the significant overlap of these distributions for the different cues strongly suggests that they cannot be directly used to determine cell fate. Here, we claim that cytoskeletal free energy, which gives a direct indication of the biochemical state of the cell, is a better metric to predict cell differentiation.

**Figure 8.**
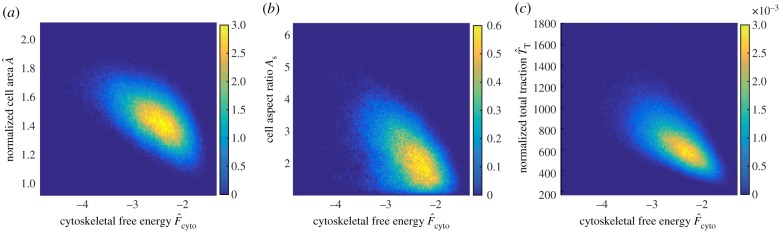
Predictions of the joint probability density distributions of the cytoskeletal free energy ${\hat{F}}_{\mathrm{cyto}}$ with the (*a*) normalized cell area $\hat{A}$, (*b*) cell aspect ratio *A*_s_ and (*c*) the normalized total traction ${\hat{T}}_{T}$. Results are shown for hMSCs seeded on an elastic substrate of stiffness $E_{\mathrm{sub}}=30\;\;\mathrm{kPa}$. (Online version in colour.)

One way to directly show why simple observables are insufficient is to plot the predictions of the correlation between common observables (i.e. cell area $\hat{A}$, aspect ratio *A*_s_ and total traction $\hat{T}$) and the cytoskeletal free energy ${\hat{F}}_{\mathrm{cyto}}$. The joint probability distributions of ${\hat{F}}_{\mathrm{cyto}}$ with each of these observables, i.e. $p(\hat{A},\;{\hat{F}}_{\mathrm{cyto}})$, $p(A_{s},\;{\hat{F}}_{\mathrm{cyto}})$ and $p(\hat{T},\;{\hat{F}}_{\mathrm{cyto}})$, are shown in figure 8*a–c* for hMSCs seeded on an elastic substrate of stiffness $E_{\mathrm{sub}}=30\;\mathrm{kPa}$. These distributions are an alternative way of showing scatter plots typically used to judge correlation of variables with the yellow regions indicating regions of high probability (i.e. if observations were made, we would anticipate to obtain a large number of independent measurements in those regions) and dark blue indicating regions of low probability (i.e. we would expect that the probability of making a measurement in these regions is small). Clearly, there seems minimal correlation between these observables and ${\hat{F}}_{\mathrm{cyto}}$ confirming our view that these direct observables are not adequate to predict hMSC commitment.

**Figure 9.**
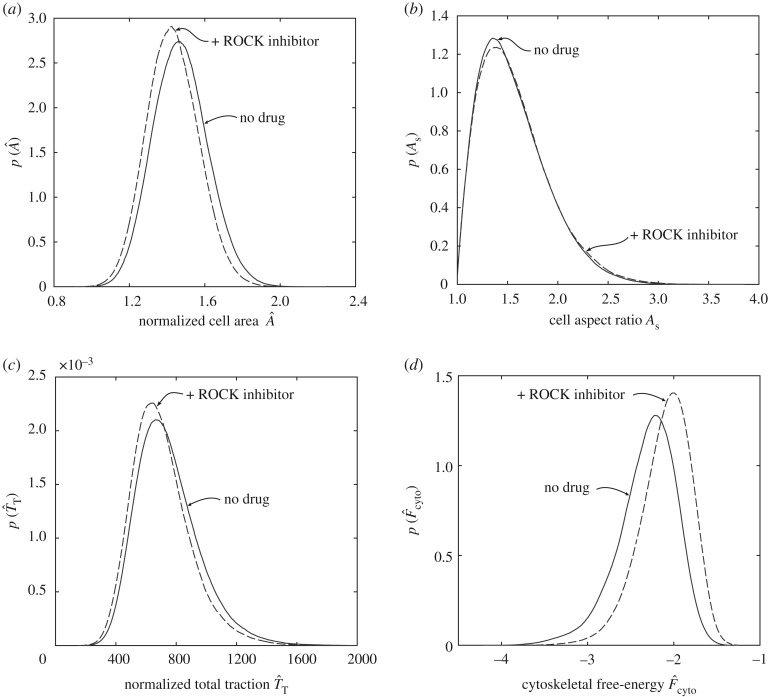
Predictions of the probability density functions for (*a*) normalized cell area $\hat{A}$, (*b*) cell aspect ratio $A_{s}$, (*c*) normalized total traction ${\hat{T}}_{T}$ and (*d*) the cytoskeletal free energy ${\hat{F}}_{\mathrm{cyto}}$ for hMSCs seeded on substrates patterned with adhesive islands of area $A_{p}=2725\;\;\mu m^{2}$. Results are shown for both untreated cells and cells treated with a ROCK inhibitor.

**Figure 10.**
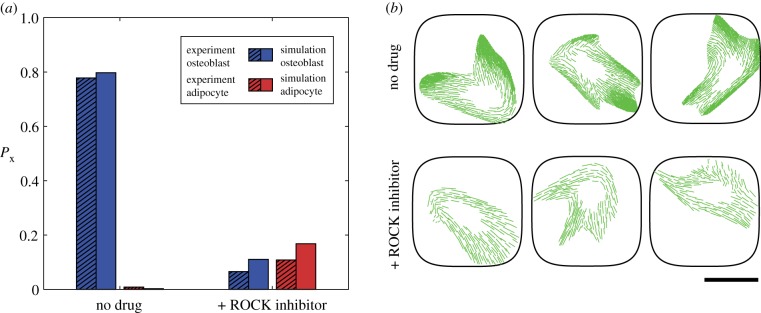
(*a*) Predictions of the differentiation fraction $P_{x}$ for hMSCs seeded on a substrate patterned with adhesive islands of area $A_{p}=2725\;\;\mu m^{2}$. Results are shown for untreated cells as well as cells treated with a ROCK inhibitor, along with the corresponding measurements from McBeath *et al*. [3] where Y-27632 was used as the ROCK inhibitor. (*b*) Three randomly selected cell morphologies from the entire homeostatic ensemble for untreated cells and cells treated with a ROCK inhibitor seeded on a substrate with $A_{p}=2725\;\;\mu m^{2}$. Scale bar, 30 µm. In these images, we only show the stress-fibre distributions to illustrate the reduction in the level of stress-fibre polymerization due to the treatment with a ROCK inhibitor. (Online version in colour.)

To further illustrate that direct observables such as cell shape or tractions may not be sufficient to predict cell differentiation, we computationally performed an inhibition study whereby we constrained cytoskeletal tension generation. This is an attempt to simulate drugs such as the Rho kinase (ROCK) inhibitor Y-27632 that restricts myosin activation. We simulated this by reducing the maximum tensile stress $\sigma_{\max}$ generated by a stress fibre from 240 to 231 kPa. Predictions of the distributions of $\hat{A}$, $A_{s}$ and ${\hat{T}}_{T}$ for cells cultured on a substrate patterned with adhesive islands of area $A_{p}=2725\;\;\mu m^{2}$ are included in figure 9*a–c* for both the reference (untreated) case and with the simulated ROCK inhibitor. There is no appreciable difference in these direct observables. The corresponding predictions for the differentiation fraction $P_{x}$ for both the untreated and ROCK inhibitor-treated cases are included in figure 10*a*. The untreated cells are predicted to show a strong commitment for osteoblasts, while the ROCK inhibitor-treated cells are predicted to display a much weaker tendency to differentiate, and also predicted to be equally likely to differentiate into adipocytes and osteoblasts. These findings are consistent with the measurements of McBeath *et al*. [3], who performed drug inhibition studies specifically to experimentally test whether cell shape affects cell differentiation. In particular, they cultured hMSCs in mixed media in the presence of 10  μM Y-27632. Similar to our computational results, they observed that the treated cells remained spread and morphologically similar to the untreated cells but no longer exhibited the differentiation response of the untreated cells. Their measurements of the differentiation fractions for cells cultured on $A_{p}=2725\;\;\mu m^{2}$ islands are included in figure 10*a*, and show excellent agreement with the computational results.

The question arises as to why the computational model predicts such a dramatic change in the response, given that common observables such as cell shape and tractions are seemingly unaffected when simulated with a ROCK inhibitor. Of course, differentiation in the model is directly related to the cytoskeletal free energy ${\hat{F}}_{\mathrm{cyto}}$. Simulating a ROCK inhibitor by reducing the value of $\sigma_{\max}$ from 240 to 231 kPa does not affect the cell morphology and tractions substantially but does affect the state of the cytoskeleton and thereby ${\hat{F}}_{\mathrm{cyto}}$, as seen in figure 9*d*. In terms of direct observations, this will be seen as a reduction in the level of stress-fibre polymerization in imaged cells. Three randomly selected cell morphologies (from the entire computed distribution of states the cells assume) for untreated and ROCK inhibitor-treated cases are shown in figure 10*b* for hMSCs seeded on substrates with $A_{p}=2725\;\;\mu m^{2}$. In these images only, the stress-fibre cytoskeleton has been marked in green similar to immunofluorescence imaging of actin in experiments. There is a clear reduction in the level of stress-fibre polymerization due to the addition of the ROCK inhibitor in line with the observations of McBeath *et al*. [3]. It is this reduction in the level of stress-fibre polymerization that enhances ${\hat{F}}_{\mathrm{cyto}}$ (figure 9*d*) and inhibits cell differentiation even though there are no major changes in the cell morphometrics. The fact that no single cell morphometric is found to correlate with the lineage hMSCs adopt is well established with learning type models, in which using multiple metrics typically found to give reasonable predictions of cell fate [22,23]. For example, it is conceivable that a combination of morphometrics such as cell area, aspect ratio and traction will correlate with cell fate for untreated cells. However, it is clear that for cells treated with a ROCK inhibitor, the metrics will need to include a quantification of stress-fibre polymerization. By contrast, cytoskeletal free energy which directly measures the biochemical state of the cell is a single metric that correlates with the lineages hMSCs adopt. Unfortunately, while ${\hat{F}}_{\mathrm{cyto}}$ is not directly measurable in experiments, the fact that such a correlation might exist provides insight into the regulatory mechanisms that govern the commitment of hMSCs.

In summary, we hypothesize that the shape fluctuations of hMSCs in response to physical cues in their microenvironment during 1–2 days after seeding determine the probability and phenotype of cell lineage commitment and subsequent differentiation. The cell shape fluctuations are an output of the homeostatic mechanics framework, with the physical cues and a simple free energy model for the cell as the only inputs. Analysed through the lens of a single biochemical parameter, i.e. ${\hat{F}}_{\mathrm{cyto}}$, the aggregate of cell shape fluctuations in a given microenvironment can provide an early forecast of stem cell differentiation (within 1–2 days, compared to the typical forecast timescale of one to two weeks in experiments [3,6]). The efficacy of the framework is demonstrated here under different conditions (mechanical and geometric cues, and in the presence of actomyosin inhibitors), thus inspiring confidence in its applicability to other insoluble cues in the stem cell niche.

## Supplementary Material

Supplementary Information

## Data accessibility

This article has no additional data.
